# Psychological Reserve/External Psychological Control in Psychotherapy: Review and New Models

**DOI:** 10.3390/bs16040485

**Published:** 2026-03-25

**Authors:** Gerald Young, Noah van Dongen

**Affiliations:** 1 Department of Psychology, Glendon College, York University, 2275 Bayview Ave., Toronto, ON M4N 3M6, Canada; 2 Department of Psychology, Erasmus University, Rotterdam Burgemeester Oudlaan 50, 3062 PA Rotterdam, The Netherlands; n.n.n.vandongen@essb.eur.nl

**Keywords:** psychotherapy, change mechanism, psychological reserve, external psychological control, internal psychological control

## Abstract

This article is about the concept of external psychological control (having a sense of psychological control over the environment). This article examines the construct of external psychological control and differentiates it from similar concepts, showing its unique attributes and how it can help understand a diverse array of psychological phenomena. Of note, external psychological control differs from internal psychological control, which is about maintaining internal psychological equilibrium or being grounded. The term psychological control is similar to terms, but we show that it differs from them and is unique in how it is presently defined. Some of these terms include distress tolerance, self-efficacy, and many variations in terms of control as part of the terminology. Another term examined is related to psychological reserve, the depletion of which affects psychological control. The article presents an original questionnaire that could be used to research external psychological control. The article concludes with a formal mathematical representation of the interaction between psychological control and psychological reserve, and with simulation research that serves as a proof of concept of the constructs proposed. The concepts of psychological control and psychological reserve can help in understanding psychotherapeutic change mechanisms. The concepts of psychological control and psychological reserve are relatively novel terms that can help understand reactions to stress and consequent stress management.

## 1. Background

The present article introduces the terms psychological reserve and psychological control. Psychological control is typically used for specifying parental control, but it has a different meaning in the present context. First, it refers to external psychological control or having a sense of psychological control over the external environment. Second, it refers to internal psychological control or maintaining equilibrium and staying grounded. The third article in the series examines the interaction of external psychological control and internal psychological control in daily functional adaptation, for example, in dealing with ongoing sociopolitical stressors. The concept of external psychological control was introduced by Young (2022b), and he applied it to psychotherapeutic change mechanisms.

This present article defines crucial terms and shows their distinction relative to related terms. For example, there are many similar terms that include the term of control. The article provides a brief questionnaire that can be used to explore the concept of psychological control in research. In addition, it includes an appendix with a mathematical representation of the main concepts under discussion.

The concept of external psychological control by itself is novel to the field and deserves the elaboration offered in the present article. Differentiating psychological control into its external and internal psychological control components further strengthens the novel conceptual base of the article, and moreover, adding the novel term of psychological reserve further points to its unique contribution in present model building. The comparison undertaken herein of psychological control with any other term that might be similar to it, no matter how remotely possible (e.g., listing of any variant term with the word control included in it), sharpens its standing as unique to the literature. Therefore, the present article consists of a narrative literature review and synthesis leading to theory building and model integration, without an empirical investigation to support it. That step in the validation of the present modeling should be the next step in testing the present concepts, and the limitations section of the article explains this further. That said, the article ends with a proposal for scale development. To conclude, the introduction of the article is on its conceptual underpinnings, the methods relate to the details of the literature search, the results concern the conceptual products rather than empirical results of a research study, and the conclusions speak to the conceptual limitations and future research directions.

## 2. Key Concepts

### 2.1. Concepts

The concepts of psychological reserve and psychological control are similar to but different from related ones. For example, as introduced to the psychological literature and defined by Young (2022b), psychological reserve is a proximal state of collected resilience factors (e.g., good sleep, good coping mechanisms) (a) that can act to buffer the effects of stress, problems, challenges, and so on, but (b) that can also be reduced or depleted by a host of factors that can lower resistance to the effects of stress, problems, challenges, and so on. This concept of psychological reserve is related to that of distress tolerance (Veilleux, 2022), as shall be shown below, but is different from it.

The concept of psychological control is defined in this present article in ways that have not been used before in the literature. As elaborated below, it concerns one’s sense of control over one’s context, or of being overwhelmed, which stands in contrast to other approaches, for example, of having a lack of control over one’s bodily response as in having a panic attack (e.g., Quagliato et al., 2021).

The concepts of psychological reserve and control are part of a suite of concepts related to a proposed integrated model of change mechanisms in psychotherapy, referred to as a biopsychosocial change mechanism model of psychotherapy. Young (2022b) developed this model to provide psychotherapists with a better understanding of the multiple avenues of change processes that could be at work in psychotherapy, and the different levels involved, for example, from more biological to more psychological (e.g., controlling bodily reactions, controlling thought processes, respectively). The model integrates the diverse set of mechanisms found in psychotherapy research on maltreatment/trauma into one overall biopsychosocial framework that can be used as a map for psychotherapy for any patient (to select what might work).

Next, this article defines in more depth novel terms related to this project of integrating psychotherapy in a causal nexus. It focuses on psychological reserve and psychological control.

### 2.2. Psychological Reserve

This elaboration of the description of psychological reserve found in Young (2022b) considers it the buffer zone people create between themselves and stress/adversity, etc., and in this regard, poor lifestyles and poor stress management/coping mechanisms reduce it. Psychological reserve is a reservoir of mental, cognitive, emotional, motivational, and stress management/coping space that is available for use in confronting ongoing and new stressors, challenges, or difficulties. It is a fluid reservoir that is depleted or boosted by the ongoing situation in these regards and the factors that continuously affect it. Psychological reserve is a phasic state influenced by multiple factors. It does not refer to trait factors, such as resilience or hardiness.

Psychological reserve is an immediate proximate factor in behavioral expression that determines response to stress, challenges, difficulties, etc. It varies with (a) physiological variables—sleep, fatigue, metabolic equilibrium (e.g., proper eating), etc.; (b) a balanced lifestyle; (c) context, e.g., ongoing stresses/challenges/difficulties and ongoing activities/functionality; (d) extant coping skills and problem solving skills; (e) traditional variables of executive function and emotional regulation as well as self-control and related mediators acting on psychological reserve; (f) personal variables, such as motivation, intelligence, and personality that might help (or not); and, (g) available material, social, and other resources and the skills in acquiring them. (h) It also varies with ongoing psychiatric state, such as anxiety, depression, irritability, PTSD, and fear-related symptoms or diagnoses, and more serious psychiatric disorders (e.g., mania, schizophrenia, personality disorder).

Psychological reserve is a concept that is broader than those that focus just on resources (e.g., Hobfoll et al., 2018), although their reference to exhaustion leading to depletion in adaptation fits current conceptualization. The concept of psychological reserve is different from those related to self-regulation and the like (e.g., Benight et al., 2018 self-regulation shift theory), because it refers to the packet of psychological mediators that allow (precede) successful self-regulation to stressors/trauma, or not. Psychological reserve can be increased by a healthier lifestyle, aside from learning how to manage stress and deal with problems more effectively, and should be a target of psychotherapy intervention beyond traditional techniques.

### 2.3. Psychological Control

Psychological control can be defined as the sense the person has of being able to manage, have agency in, and otherwise not be overwhelmed by the context in which one is adapting functionally (or the sense that it can be regained/improved when compromised/lost). Granted, sometimes people have a too-rigid sense of psychological control, and it extends into obsessive/compulsive behavior, dysfunctionally avoiding required flexibility in behavior, emotion, and thought, etc., such as in avoiding adaptive risk taking, when required. Or, people greatly limit the areas to which they seek psychological control, having experienced difficulties in other areas of their life or in the past. Or, they have only minimal control, and in a limited range of domains, and any disruption in these limited domains becomes quite serious for them, no matter how limited. However, in the approach herein, we deal with the generic sense people have or desire to take back (to the degree possible) psychological control after ongoing stress and experienced trauma. In the broadest sense, as defined here, psychological control also refers to subordinate mechanisms, such as coping skills and emotional regulation. As well, it is consistent with other psychological constructs, such as executive function.

## 3. Terminology Related to Psychological Reserve and Psychological Control

### 3.1. Psychological Resource Model

The figure indicates the network of concepts related to but different from the novel concepts of psychological reserve and psychological control. The right side of the figure indicates the range of distal and proximal factors that determine whether the individual will deal with ongoing stress/problems/challenges with a flexible, adaptive executive control and problem-solving. Psychological reserve constitutes a depletable (or boostable) state or reservoir of psychological factors that can affect ongoing adaptive behavior, including one’s perception that the present context is overwhelming, or rather, perceived as allowing (continued) sense of having psychological control. The left side of the figure indicates contributing factors to the variability in the state of psychological reserve and having a sense of psychological control. These factors are different from them and should not be confused with them. These factors typically are considered traits, and are less influenced by other ongoing factors, but can vary in state according to them to some degree. Resilience refers to both the process and outcome of effort to flexibly adapt to problematic or challenging life contexts, and varies in degree of success. It is accompanied by risk factors that can act to reduce the adaptive accommodations (e.g., APA, American Psychological Association, 2015). Diatheses refer to the differential vulnerabilities for a particular psychiatric condition, as well as the interactive stress that triggers vulnerability. Individuals vary in the vulnerability and the stresses related to them, such that their interaction determines the outcome involved due to the vulnerability (partly after Broerman (2018)). Distress tolerance refers to one’s perceived capacity to “endure and cope with” negative or uncomfortable emotional experiences (R. J. Brown et al., 2022). The APA dictionary (American Psychological Association, 2015) adds that it relates partly to the emotions in pursuing desired goals or maintaining goal-directed behaviors when in distress. It has been included as one component in dialectical behavior therapy (DBT). Psychological co-regulation is a term developed by Young (2022a) to encompass emotional, cognitive, self, behavioral, and related regulations. It refers to the degree possible, considering the range of factors that can influence it, in the modulation/effort to control, and optimization of the desired outcome in context, as well as presentation of oneself in context. Psychological regulation is exquisitely interactive and not a property of the organism displaying it. It can refer to “lower” organisms as well. In this sense, a broader term for it is “relational co-regulation.” Decisional control “entails predictive judgments surrounding the amount of threat associated with alternatives embodied by the presenting stressor environment” (Neufeld, 1999, p. 388). Locus of control refers to the differential perception of the control one has over one’s life course, present circumstances, and conditions/outcomes. It includes one’s motivational orientation to one’s ongoing life course and those circumstances and conditions. If one’s life course is categorized as “external,” the perception is that external factors out of their control govern their lives/course, circumstances/conditions/outcomes. In contrast, those having an internal locus of control relate their lives and course/circumstances/conditions/outcomes to their own agency and abilities (adapted from the APA Dictionary, 2015). In the present author’s view, the overcontrolling behavior/attitude involved applies to the full range of components in behavior (affective, cognitive, corporal, etc.) and control of the other (person), as well. Resource models related to stress/problem/adaptation management will refer to the person’s resources, including personal (e.g., coping, motivation), and social (even therapeutic) ones, or their lack. The variables on the left and right of the figure can vary in degree and serve to amplify, dampen, or moderate psychological reserve, psychological control, and outcomes in flexible adaptivity.

### 3.2. Elaboration

Veilleux (2022) described a psychological resource model applicable to the concept of distress tolerance that speaks to the present conceptualization of change mechanisms in psychotherapy and reinforces the present approach as a valid one. Distress tolerance refers to (a) the perceived capacity to withstand distress, whether physical or emotional, and (b) the behavioral efforts used to persist through the distress being experienced. The distress could be emotional, cognitive, or physical. It could be from actual events, recalling past events, or anticipating future events.

As for her model of distress tolerance, it considers susceptibility to being overwhelmed by distress in terms of proximal, immediate, momentary factors in one’s psychological resources. These include fatigue/hunger/illness internally and social support/being alone externally. Psychological resources also include intrapersonal and cognitive factors (e.g., confidence, problem-solving skills).

First, comments note that Veilleux’s (2022) concept of psychological resources as mediating distress tolerance is highly similar to the present concept of psychological reserve. Also, the concept of psychological reserve compared to the concept of psychological resources implies that the contents are ready for deployment and can act or not act as buffers, while the concept of psychological resources appears too generic to give that connotation in all cases. Second, according to Veilleux (2022), traits do not “cause” people to do things, but we disagree with that. Traits are higher-order “overarching” patterned actions and mental habits and, in Young’s (2015, 2023a, 2023b) hybrid network-systems approach to behavioral influences and organization, top-down factors, such as traits, can influence bottom-up, lower-order factors, such as behaviors and symptoms, as much as the reverse direction taking place, in a reciprocal model (for a similar model, see O’Driscoll et al., 2022). Additionally, Veilleux’s model does not include the ultimate intervening factor that appears to develop according to the present model. That is, one’s sense of psychological control appears to be the most immediate, proximal mechanism concerning adaptivity/maladaptivity to the environment, and distress tolerance is just one factor related to it that can influence it. Note that distress tolerance has been related to PTSD, which is a primary focus of the type of distress under discussion (Akbari et al., 2022).

### 3.3. Other Terms

#### 3.3.1. Introduction

**Figure 1:**
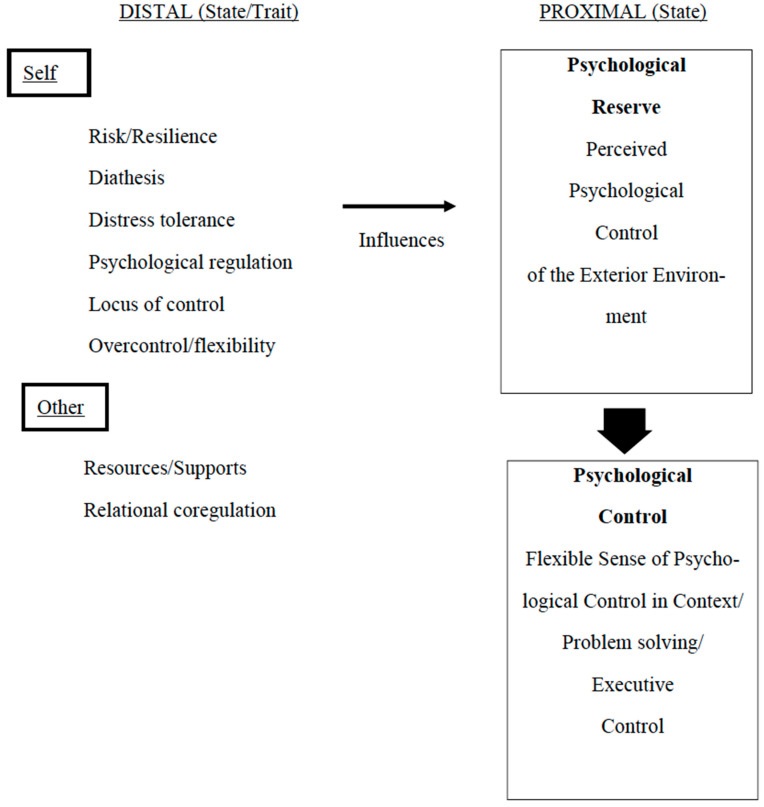
Nexus of psychological reserve/control concepts.

Figure 1 differentiates the novel concepts of psychological reserve and psychological control from similar but different ones. For example, psychological reserve is a variable state reservoir to help buffer stress and the like, and the other similar terms in the figure represent factors that could influence it but do not define it.

This completes the review of key terms in the article. Next, an extensive text lists all concepts related to psychological control, with the goal of informing how it brings added value to the field.

#### 3.3.2. Methods

The present article conducted an extensive PsychInfo search in November 2025, in order to present tabular definitions and comparisons of key terms. The goal was to show the unique contribution of the concepts of psychological reserve and psychological control to the literature on psychotherapy change mechanisms. For each term in the table of key terms, at least one recent article in the PsycInfo search that best fit the needs of the present article was chosen for inclusion. Some terms related to the concept of the psychological control approach its meaning and also help clarify its unique difference.

#### 3.3.3. Psychological Control and Psychological Reserve: Definitions and Comparison

**Psychological Control** (per the present article) is the sense the person has of being able to manage, have agency in, and otherwise not be overwhelmed by the context in which one is adapting functionally (or the sense that it can be regained/improved when compromised/lost). Trauma reactions are potentiated when a sense of psychological control over the environment is lost because of overwhelming external events. There are tens of related terms mentioned herein, but the definition stands out as indicating the maintenance or regaining of an internal equilibrium state related to a disruptive, external, overwhelming event. Individuals seek psychological control in the sense of emotional regulation internally, but also in the sense of not being overwhelmed by external events or repairing when overwhelmed.

**Psychological Reserve** (per this article; originally defined in Young (2022a)). Psychological reserve is a proximal state of collected resilience factors (e.g., good sleep, good coping mechanisms) (a) that can act to buffer the effects of stress, problems, challenges, and so on, but (b) that can also be reduced or depleted by a host of factors that can lower resistance to the effects of stress, problems, challenges, and so on. Psychological reserve resembles other factors, such as cognitive reserve, ego depletion, etc., but differs because both internal states and external factors can reduce or raise it. It differs from these other terms by its psychological emphasis and its effects on stress management and having a sense of control.

**Uncontrollability** (from Şener and Altan-Atalay (2025)). In Şener and Altan-Atalay (2025), uncontrollability referred to the lifetime perception of early- and later-life experiences and stressors. The manner in which uncontrollability is used presently in the concept of psychological control relates to perception of the overwhelming nature of current events—such as in trauma reactions—rather than past ones, and does not imply that past events are cumulative.

**Locus of Control** (per this article; adapted from Foster et al. (2025), who referenced Duttweiler’s (1984) work). Internal compared to external locus of control is defined in terms of whether the person believes that they have control over their lives (rewards and punishments are contingent on their own actions) and not external events, fate, etc. (unlike in external locus of control). Locus of control is not a uniform concept in control that applies the same way to trauma victims, nor helps understand the present concept of psychological control, but it could be one factor that moderates its expression.

**Sense of Control** (adapted from Garner et al. (2025), citing Lachman and Weaver (1998b)). Concerns arise when an individual perceives that their goals and desires are influenced by factors beyond their control. The concept is related to personal life constraints, not events at hand. Sense of control, as defined, does not relate to an external event that is overwhelming, so it does not approximate psychological control as defined in this manuscript.

**Anxiety Control** (adapted from Senger et al. (2025), citing T. A. Brown et al. (2004)). Perceived control over anxiety-related events reflects one’s sense of personal agency related to the event(s) in question, owning one’s emotions, and how one is managing the stressor. The construct is more related to agency, emotion, threat, and stress management rather than psychological control, as herein defined.

**Fear of Losing Control** (adapted from Radomsky and Gagné (2019), see Khosravani et al. (2025)) refers to the angst of losing control over one’s thoughts, feelings, behavior, and physical reactions, leading to catastrophic consequences. The Beliefs About Losing Control Inventory (BALCI) has been found to have two major factors: negative beliefs about losing control (I’m afraid of losing mental control; paraphrased) and the importance of staying in control (I need to stay in control mentally). Similar constructs include the desire for control, unrealistic control beliefs, affective control, and anxiety sensitivity. The contents of the BALCI and associated scales are more about self-controlling one’s thoughts, etc., and not external events, unlike in psychological control.

**Life Control** (adapted from Heise et al. (2025), citing Kerns et al. (1985)). West Haven–Yale Multidimensional Pain Inventory (WHYMPI) Section A yields subscale scores for life control, among others. The scale is used in chronic pain assessments. Chronic pain that is associated with disability can be subjectively evaluated as a loss of control over one’s life. The precipitating event is an internally experienced pain, which is different than the present concept of psychological control.

**Personal Control** (adapted from Xu et al. (2024), citing Kay et al. (2008, 2009)). The person’s belief that they can personally predict, affect, and steer events in the present. When it is diminished or threatened, anxiety can result. Personal control is a belief that can influence one’s sense of psychological control, but the concept lacks the emphasis on the perception of an overwhelming event.

**Trait Self-Control** (adapted from Zhang et al. (2023), citing de Ridder et al. (2012), Tangney et al. (2004)) is the trait-like ability to override dominant responses. It might be a factor in the ability to deploy psychological control.

**Overcontrol** (adapted from Steinhoff et al. (2025), citing, e.g., Lynch (2018)). A set of characteristics that includes high perfectionism, cognitive inflexibility, and a desire for control. It might be a factor in the ability to deploy psychological control.

**Control Processes** (adapted from Debra et al. (2024)) refer to affective inhibition to regulate emotional reactivity, with heart rate variability as an index. It might be a factor in the ability to deploy psychological control.

**Internal Control** (adapted from Sayar and Çapik (2025)) is the same as the internal locus of control. See the comments above.

**Compensatory Control** (adapted from Fritsche et al. (2025)) refers to group-based (e.g., sociopolitical) processes of re-establishing control. The person can engage in personal agency but will revert to group identity as required. It is unrelated to the present concept of psychological control.

**Distress Tolerance** (per this article, adapted from Veilleux (2022) and Lopez et al. (2024)) refers to perceived capacity to withstand distress, whether physical or emotional, as well as the behavioral efforts used to persist through the distress being experienced. The distress could be emotional, cognitive, or physical. It could be from actual events, recalling past events, or anticipating future events. Relative to psychological control as presently defined, distress tolerance is a broader concept because it includes reactions to possible and past events in addition to actual events. Lopez et al. (2024) found that it is related empirically to Neuroticism. Moreover, it refers to the capacity to cope with distress, rather than losing psychological control and then regaining it. Like for self-efficacy, it could be a factor that contributes to psychological control.

**Self-Efficacy** (adapted from Littleton et al. (2025), Murphy et al. (2025), citing Bandura (1977)). Coping (with trauma) self-efficacy includes beliefs about one’s ability to cope with traumatic events and areas of one’s life affected by the trauma reactions that have taken place as a mechanism of posttraumatic recovery. Self-efficacy concerns the belief that one can maintain an internal equilibrium state in relation to an overwhelming external event. It can be one factor in regaining a sense of psychological control after a perceived overwhelming event.

**Emotion Regulation** (adapted from Clinchard et al. (2025), citing Gross et al. (2006)). Constructive emotional regulation involves cognitive reappraisal (interpreting the emotion-eliciting situation to reduce its emotional impact), whereas expressive suppression involves inhibiting ongoing emotion-expressive behavior. Once more, internal self-management cognitively can help modulate trauma reactions to overwhelming external events, helping in regaining a sense of psychological control as a contributing factor; however, it is not a control variable per se.

**Personal Mastery** (adapted from Spooner et al. (2025), citing Lachman and Weaver (1998a)). The Personal Mastery and Perceived Constraints scales (Lachman and Weaver, 1998a) measure the individual’s beliefs about the control that they have in a given situation. As discussed for sense of control above, the Lachman and Weaver concept of control does not relate directly to the present one of psychological control.

**Ego Depletion** (adapted from Baumeister et al. (2024)). Ego depletion theory has extended beyond its original conceptualization to include emotion regulation effects not from depletion of willpower with successive activity per se but from efforts to conserve remaining energy. The depletion/conservation of the reserve affects decision making, rational, intelligent thinking, planning, and so on. Willpower is critical to adapting to stress, trauma, etc. Maintaining/regaining a sense of psychological control of overwhelming external events depends on the desire to deal with them. But willpower by itself is not a psychotherapeutic change mechanism, although it is an influencing factor. Its depletion/maintenance is critical, for example, related to emotion regulation, but the present concept of psychological reserve is a broader one than efforts to conserve energy for willpower.

**Tolerance of Uncertainty** (adapted from Sahib et al. (2023), citing Carleton (2016, p. 31)) is the individual’s dispositional incapacity to endure the aversive response triggered by the perceived absence of salient, key, or sufficient information, and sustained by the associated perception of uncertainty. Dispositions can serve as generalized influencing factors on the specific change mechanisms applied in psychotherapy, but are not change mechanisms themselves.

**Learning/Conditioning/Extinction.** Exposure therapy works toward the extinction of the fear response, counterconditioning, and learning more adaptive responses. Learning is a general change mechanism transdiagnostically. But targeting relearning or different learning might work best when more basic change mechanisms are targeted, such as increasing one’s sense of having psychological control. Better learning and better psychological control can work reciprocally to improve psychotherapeutic outcomes.

**Cognition/Metacognition/Expectancy** (adapted from Pukstad et al. (2025), citing Wells and Matthews (1994, 1996)). Aside from its focus on earning, exposure therapy works at cognitive levels to restructure cognitions and narratives. Altering negative expectancies is critical. Dysfunctional metacognition beliefs involve beliefs about thinking and cognition, including the need to control thoughts (e.g., “If I did not control a worrying thought, and then it happened, it would be my fault”). These types of generic statements on domains of psychological function that need to be improved in psychotherapy do not indicate change mechanisms per se, but the adjunct psychological domains that could be targeted in therapy. Meta-cognitive beliefs include the need to control thoughts, which is an internal process, unlike the control referenced in psychological control (about overwhelming events).

**Motivation** is critical to regulating stress response and dealing with stressors, problems, and challenges that activate stress response. Motivation is not a change mechanism per se, but the therapist acts to increase it as a mediator in empirically supported mechanisms of change.

**Hope** (adapted from Long et al. (2025)). CBT that instills hope is beneficial for anxiety and depression. Hope can be involved in the pathways that increase a sense of psychological control, altering perceptions of the index event.

**Conservation of Resources** (adapted from Burton et al. (2025, pp. 2–3), citing Hobfoll (2011)). Individuals manage stress through the acquisition, preservation, and protection of valuable resources: personal, social, material, and energy. [R]esource losses can cascade, amplifying psychological distress, while resource gains promote resilience. This stress management model speaks to the present concept of psychological reserve, which is embedded in the personal domain of Hobfoll’s resource model. His model emphasizes the resource of mental health, while the present model speaks to the depletion in psychological reserve that can lead to a decline in mental health.

**Reserve Capacity** (adapted from Garcia et al. (2025), citing Gallo and Matthews (2003)). Psychosocial resources promote health and buffer against the negative impacts of stress. People can call upon their psychosocial resources (tangible, interpersonal, intrapersonal, or cultural) in times of stress, including intrapersonal optimism and perceived control. Reserve capacity approximates the present concept of psychological reserve, but it emphasizes psychosocial factors, which are only one aspect of psychological reserve. Reserve capacity can include calling on psychological control, but in the present concept, psychological control is affected by psychological reserve depletion.

**Cognitive Reserve** (adapted from Fares-Otero et al. (2024), citing Kolb and Gibb (2011)) is the individual’s ability to use efficiently and flexibly available cognitive skills and networks to adapt to the environment. The concept is tied to available brain resources, as in dealing with dementia. Psychological reserve refers to the degree of available resources, cognitively, emotionally, and physically, that are available to resist stress, overwhelming external events, and so on. However, cognitive reserve is just one aspect.

#### 3.3.4. Comment

The present section of the article that lists every possible term related to psychological control and psychological reserve serves to underscore their original status in the field. Most of the competing terms involve the word “control,” so they are appropriate candidates for this section. Others approach the conceptual basis of the terms of psychological control and reserve, so as to amplify how the proposed terms are different from the competing terms. Some of the comparable control terms are not even closely related despite the word “control” in their terminology, which is noted herein; others have some semblance of similarity on analysis, but not sufficiently to replace the proposed terms of psychological reserve and control. “Control” is a common word in psychology, and the concept of psychological control is common, as well, in the context of parenting. However, the latter use of the term is very different from the present use, and the present use of the term has withstood the comparative analysis of even remotely similar terms so that the present control terms and their definitional bases used herein stand as unique and worthy of further empirical development and investigation.

## 4. Future Research Directions

### 4.1. Questionnaire

In reviewing scales related to psychological control (in the above), it became apparent that the control-related items were not written to represent the construct of psychological control, as presented here. Therefore, the first author developed a tentative list of items that could be used to create a psychometrically valid Psychological Control Scale. The proposed scale borrows from the Distress Tolerance Scale (DTS; R. J. Brown et al., 2022), changing the term “distress or upset” to “no psychological control or feeling overwhelmed.” There are four scales in the DTS, and the brief version takes one item from each component. The four components are referred to as tolerance, appraisal, absorption, and regulation (which are related to (a) poor tolerance for feeling no psychological control or feeling overwhelmed, (b) affected appraisal of such, (c) absorption in such, and (d) poor regulation of such). The brief version items include, respectively (and modified for present purposes): (a) I can’t handle feeling no psychological control or feeling overwhelmed; (b) Having no psychological control or feeling overwhelmed is always a major ordeal for me; (c) My feelings of no psychological control or feeling overwhelmed are so intense that they completely take over; (d) I’ll do anything to stop feeling no psychological control or feeling overwhelmed.

### 4.2. Mathematical Representation

To further specify these various psychological and psychotherapeutic models of change for PCT, Kennaway (2020) described a precise model of change couched in control parameters and differential equations. Sompolinsky (e.g., Sompolinsky, 2014; Hu and Sompolinsky, 2022) has differentiated perceptual learning in terms of theoretical physics, mathematics, and computational neuroscience across scale, from neuron to neuronal circuitry to behavior, while focusing on excitation/inhibition, much like in the present modeling.

Beyond that, the work of van Dongen et al. (2025) provided a testing framework of theories referred to as “productive explanation”. Wang et al. (2024) introduced mathematical and computational modeling generally. In differential equations, the left-hand side typically represents the derivative of a variable *x* at issue (in this case, psychological control) with respect to a variable such as time t (giving *dx*/*dt*).

Using this format, the article authors created a testable model of maximal psychological control (C) and the influence of psychological reserve (R) on it. The rate of change over time (t) is indicated on the left of the equation, and the variables that can affect it as well as the products of the psychological activity involved (C, R, i = instances in time, over which different life and therapeutic events can have different negative or positive effects) are indicated on the right, along with the possible depletions (δ) and recovery (^r^) as behavior proceeds. There is a maximum C and R, indicated with *C*^r^ and *R*^r^ (set to 1), to which C and R return over time if depletion is set to zero. In this case, ρ is the recovery rate, which determines how long this recovery takes. The subscript “t” of δ indicates that the depletion rate can vary with time (instead of being a constant). The representation in the equation indicates how the ratio of psychological control and psychological reserve and the changes therein over time (depletion, recovery) determine psychological outcome, including in psychotherapy. A full technical specification of this model, including a formal definition of all variables and parameters, plausible value ranges, and reproducible R code for simulating its dynamics, is provided in Appendix A.

The formula does not consider factors such as whether the superscript “i” identifies particular periods of time where δ has a particular value (like for stages). It does not offer a separate formula for R, because it also varies with time and determines the behavior of C. The representation of the relationship between C and R as a multiplication product requires empirical testing, and they may relate otherwise. The productive explanation framework requires that these parameters and variables are given (ranges of) values. Then, data are simulated from this model and matched to existing empirical data, which could be the next steps in the theoretical modeling process. The Appendix A illustrates this workflow by showing example simulations under different depletion and recovery regimes, which can, in principle, be compared to empirical time-series data on psychological functioning and treatment response.

d*Q*/d*t* = −δ^i^_t_ Q + ρ(*Q*^r^ − *Q*), where Q = C × R (1)

The model lists the primary variables at issue, ones that serve in the mediation causally of the model, but it leaves out the multiple variables that can influence the outcome, such as moderating constraints, patient factors (e.g., age, sex, SES, co-morbidities), therapy and therapist factors (such as therapist empathy and fit, the techniques used). These factors can be organized into a biopsychosocial model and also serve for further representation and testing of the psychological control model.

## 5. Final Remarks

### 5.1. Limitations

As for the limitations of the presented model and novel terms, note that it is impossible to provide empirical support for the model at this time. Review, conceptual, and theory papers are common in psychology. They might include supportive empirical data, but not necessarily. They might refer to data collected in other studies, but not necessarily. They might be in a position to move to the next step of developing a research program in support of the proposed model, but this can take place only after the model is published and affords a basis for proceeding to study it, which is the present case. Papers based on models are supported by narrative reviews, which is the present case. They differentiate the relevant terms from competing ones, which is the present case. They present a mathematical representation of the models and simulation studies; this step prepares for explicit testing of the model proposed, which is the present case. They present templates of questionnaires that could be used to investigate the novel components of the model empirically, which is the present case. They suggest clinical applications in thorough guidelines, which is the present case. This together will lead to empirical research not only on the components of the model, for example, with clinical populations, but also on the clinical applications of the model, further supporting it; however, that lies quite far in the future, given that this publication is the first step in this typical conceptual and empirical programmatic development of models in psychology that might end up widely used and cited.

### 5.2. Directions for Future Research

Further specification of the concepts of psychological control and psychological reserve, as well as the development of questionnaires, measures, or scales that serve to quantify them, will prepare for strict empirical testing of hypotheses deriving from them. Future research should concentrate on the next steps for instrument validation, including specification of the generalization to their use with target populations, psychometric benchmarks, and differentiation of other questionnaires in the field, to determine construct overlap. The appendix has demonstrated the feasibility of this type of research program by its simulation study. The focus of future research on the two concepts of psychological reserve and psychological control, and their lower or higher levels of function, will depend on the researcher’s interests, but the range of possible topics is large. For example, one researcher might investigate the influence of these factors in the context of trauma exposure and the development of PTSD. Are the main effects by themselves more telling than the interaction effects between psychological control and psychological reserve, and what factors account for their lowering and their role in the development of PTSD, its maintenance, and its amelioration in subsequent psychotherapy? The research should differentiate between internal and external psychological control in this regard. The research design will include these control variables as dependent measures, and the outcome measures will relate to the specific hypotheses, e.g., PTSD symptom change with intervention. The research design should include measures of similar hypotheses to determine the unique role of the control variables under investigation, as determined by step-wise hierarchical multiple regression. Once empirical research is undertaken to test the present model building, it can inform the mathematical representation of the model herein, which can be subsequently revised with further, more refined simulation studies. The methodological assumptions of the mathematical representation include that the concepts of external psychological control and psychological reserve are specific enough to allow for their mathematical representation, and this formalism can help guide and structure research on the model and allow specific testing of its components for their validity. This makes the interaction between progressive mathematical representations of the evolving concepts and mathematical formalisms of the model, with ongoing empirical research testing it, vital to its validation and application in context, including clinically. Future research should concentrate on the next steps for instrument validation, including specification of the generalization to their use with target populations, psychometric benchmarks, and differentiation of other questionnaires in the field, to determine construct overlap.

### 5.3. Applications

Perceived stress/difficulties impact our psychological reserve and sense of external psychological control; when these are reduced, it is more likely that we develop psychological disorders. Psychotherapy should focus on increasing these variables in context; psychotherapy should focus on documented change mechanisms as well as those related to psychological control and psychological reserve. This approach to psychotherapy can provide an integrated perspective that can guide therapists in cases of PTSD and other conditions, such as depression and chronic pain.

### 5.4. The Validity of the Theorizing

Appendix B instantiates the differential and novel approach of the concepts of psychological control and psychological reserve as developed in the present article and the companion one (Young, 2026). This appendix has been written to respond to potential (a) general and (b) theoretical criticisms of the concepts of psychological control and psychological reserve and in their mathematical representation.

## Data Availability

No new data were created or analyzed in this study.

## Appendix A

### Appendix A.1. External Psychological Control × Reserve Model

#### Appendix A.1.1. Introduction

In this simulation, the concept of an interaction between external psychological reserve and psychological control has been modeled and subject to a simulation proof of concept. The model reflects the parameters of the concept as described in the article, and sets the stage for further model specification and simulation testing. Many factors can influence both components of the model (psychological reserve, external psychological control), but the directions for therapeutic interventions related to both components of the model have been specified in this initial dynamic modeling of the concepts. The concepts (psychological reserve, external psychological control) need further conceptual and empirical work to increase the validity of the concepts and the reliability of their application in psychotherapy. This will contribute to a better understanding of the change mechanisms in psychotherapy and their underlying dynamics. In essence, this appendix implements and explores a simple differential equation model for external psychological control and psychological reserve.

We define an effective state variable where C(t) is external psychological control and R(t) is psychological reserve. The dynamics of Q(t) are given by $Q^{r}$: resting/maximal level of effective control × reserve; ρ: recovery rate; and $\delta_{t}$: time-varying depletion rate (demands, life events, therapy, etc.).

(A1) Q(t)=C(t) × R(t),

(A2) $$\frac{dQ}{dt}=-\delta_{t}\;Q+\rho \;(Q^{r}-Q),$$

In this implementation, we model only Q(t) explicitly as a state variable; C(t) and R(t) are conceptual components whose product is represented by Q(t).

To translate this mathematical model into a simulation, we implemented the differential equation in R and solved it numerically over time using standard ODE solvers. In the code, $\delta_{t}$ is specified as a time-varying function that can change across predefined intervals (e.g., periods of stress or therapy), while ρ and $Q_{r}$ are treated as fixed parameters within chosen ranges. By assigning plausible values to these parameters (see below) and simulating trajectories of Q(t), the model produces concrete predictions about the temporal evolution of external psychological control and reserve. These simulated trajectories can, in principle, be linked to observable variables (such as symptom scores or task performance) and compared to empirical time-series data, allowing the model to be tested and refined within the productive-explanation framework.

#### Appendix A.1.2. Variables and Parameters (With Ranges and Interpretation)

Below, we summarize the model components, suggested ranges, and interpretations. You can adapt these ranges to your empirical context.

#### Appendix A.1.3. State Variable

- **Q(t)**
  ○ *Role*: State variable.
  ○ *Meaning*: Effective external psychological control at time *t*, conceptualized as the product of control and reserve, Q=C×R
  ○ *Scale*: 0–1 (proportion of “maximal” control × reserve). We think it is fair to assume that such a property has a maximum and minimum value. These might differ per person, but for simplicity, we can always transform these to 1 and 0, respectively. This applies to all 0–1 scales.
  ○ *Default initial value*: 1 (starting at full control/reserve).
  ○ *Notes*: When Q is small, either control or reserve, or both are low.

#### Appendix A.1.4. Conceptual Components

- **C(t)**
  ○ *Role*: Conceptual component of Q.
  ○ *Meaning*: Level of external psychological control at time *t.*
  ○ *Scale*: 0–1, with $C^{r}=1$ as resting/maximal control.
  ○ *Note*: In this implementation, C is not simulated separately; it is aggregated into Q.
- **R(t)**
  ○ *Role*: Conceptual component of Q.
  ○ *Meaning*: Psychological reserve (buffer, capacity) at time *t.*
  ○ *Scale*: 0–1, with $R^{r}=1$ as resting/maximal reserve.
  ○ *Note*: Also not simulated separately; R’s influence is “folded into” Q.

#### Appendix A.1.5. Parameters

- $Q^{r}$ (Qr in code)
  ○ *Role*: Parameter.
  ○ *Meaning*: Resting/maximal level of effective control × reserve ($Q^{r}=C^{r}{\times R}^{r}$).
  ○ *Typical range*: Often fixed at 1 (but could be >0 if you want individual differences in maximum).
  ○ *Interpretation*: Upper bound toward which Q recovers when depletion is absent.
- ρ (rho)
  ○ *Role*: Parameter, recovery rate.
  ○ *Meaning*: Speed at which Q returns toward its resting level $Q_{r}$ in absence of depletion.
  ○ *Units*: 1/time unit.
  ○ *Typical range*: 0.01–1.0.
    ▪ Small (e.g., 0.01–0.05): very slow recovery.
    ▪ Medium (0.1–0.3): moderate recovery.
    ▪ Large (>0.5): rapid recovery.
  ○ *Interpretation*: Higher ρ → faster recovery from depletion.
- $\delta_{t}$ (time-varying depletion rate)
  ○ *Role*: Time-varying parameter (function of t and “instances” i).
  ○ *Meaning*: Net depletion rate at time *t*, reflecting demands, life events, therapy, etc.
  ○ *Units*: 1/time unit.
  ○ *Typical range*:
    ▪ Baseline: 0–0.1 (mild day-to-day depletion).
    ▪ Stressful events: 0.2–1.0 (strong depletion).
    ▪ Optionally $\delta_{t}<0$ to represent strongly restorative conditions (net boosting), but in the basic “depletion” version $\delta_{t}\geq 0$.
  ○ *Interpretation*: Higher $\delta_{t}$ → stronger current depletion of external psychological control.
- **Event timing parameters (implementation details for** $\delta_{t}$ **)**
  ○ t_event_start, t_event_end: time window of a stressful period (e.g., major life stressor).
  ○ t_therapy_start, t_therapy_end: time window where therapy reduces depletion.
  ○ These are not “core” theory parameters, but implementation details specifying when $\delta_{t}$ changes.

#### Appendix A.1.6. Model Specification and R Implementation

This section contains the implementation of the ODE model in R, the simulations, and the resulting plots. The relevant R code is presented in the gray boxes.

#### Appendix A.1.7. ODE Model

The following chunk defines the ODE model function. It implements and allows the depletion rate $\delta_{t}$ to vary over time (baseline, stressful period, therapy).

(A3) $$\frac{dQ}{dt}=-\delta_{t}Q+\rho (Q^{r}-Q),$$

model_Q <- **function**(t, state, pars) { **with**(**as.list**(**c**(state, pars)), { *# ----- Time-varying depletion rate delta_t -----* *# Baseline depletion:* delta_t <- delta_baseline *# Stressful period with stronger depletion* **if** (t **>=** t_event_start **&&** t **<** t_event_end) { delta_t <- delta_event } *# Therapy period with reduced depletion* **if** (t **>=** t_therapy_start **&&** t **<** t_therapy_end) { delta_t <- delta_therapy } *# Differential equation:* *# dQ/dt = -delta_t * Q + rho * (Qr - Q)* dQ **<-** **-** delta_t ***** Q **+** rho ***** (Qr **-** Q) *# Return derivative and also delta_t as an output variable* **list**(**c**(dQ), delta_t = delta_t) }) }

#### Appendix A.1.8. Default Parameters and Single Simulation

The next chunk sets default parameter values (recovery rate, depletion levels, and event timings), defines the initial state, and runs a single simulation of the model over time using ode().

*# Default parameters* pars <- **c**( *# Core theory parameters* rho = 0.20, *# recovery rate* Qr = 1.00, *# maximum/resting level* *# Depletion parameters* delta_baseline = 0.05, *# baseline depletion (day-to-day demands)* delta_event = 0.50, *# depletion during stressful period* delta_therapy = 0.02, *# reduced depletion during therapy* *# Event timing (in arbitrary time units)* t_event_start = 10, t_event_end = 30, t_therapy_start = 30, t_therapy_end = 60 ) *# Initial state and time grid* state <- **c**(Q = 1.0) *# start at full control × reserve* times <- **seq**(0, 100, by = 0.1) *# simulation horizon* *# Run model* out <- **ode**(y = state, times = times, func = model_Q, parms = pars) out_df <- **as.data.frame**(out) **head**(out_df) ## time Q delta_t ## 1 0.0 1.0000000 0.05 ## 2 0.1 0.9950617 0.05 ## 3 0.2 0.9902454 0.05 ## 4 0.3 0.9855480 0.05 ## 5 0.4 0.9809669 0.05 ## 6 0.5 0.9764988 0.05

#### Appendix A.1.9. Plots for a Single Parameter Setting

The next two chunks create plots of the simulated trajectory of Q(t) and the corresponding time-varying depletion rate $\delta_{t}$.

**ggplot**(out_df, **aes**(x = time, y = Q)) **+** **geom_line**(linewidth = 1) **+** **labs**( x = "Time", y = "Q(t) = C(t) × R(t)", title = "Dynamics of psychological control × reserve" ) **+** **ylim**(0, 1.05) **+** **theme_minimal**()

**ggplot**(out_df, **aes**(x = time, y = delta_t)) **+** **geom_step**(linewidth = 1) **+** **labs**( x = "Time", y = **expression**(delta[t]), title = "Time-varying depletion rate" ) **+** **theme_minimal**()

## Appendix B

### Appendix B.1. Creating and Testing Empirically Psychological Models: The Case for Psychological Control and Psychological Reserve

General Critique

See Section 5.4

### Appendix B.2. The Construct

The construct of psychological control has been demonstrated as relatively novel by a comparative analysis that sets boundary conditions. The relevant concepts are differentiated according to their epistemological status and whether they are mere formalisms or represent actual psychological function, in addition to locating their primacy among other concepts.

Psychological constructs can vary in a number of ways. They can be heuristic concepts that are not conceived of as actual, active psychological entities. Perhaps they are reasonable proxies to what might be occurring psychologically, but that is a supposition that would require empirical investigation. Psychological constructs can be mathematical representations not meant to represent actual, active psychological entities. They are mere formulations to spur further conceptualization and investigation. Each of the latter options—the heuristic and the formulaic—does not approach effectively by itself actual active psychological entities. Psychological constructs might be posited to represent actual, active psychological entities that have real-world and practical implications and applications. However, they would need to make further additions to confirm that possibility. First, they would need empirical investigation to support their validity. Second, they would need an understanding of the underlying mechanisms involved in their expression and modulation. Additionally, the influences in both senses on the expression and modulation of the construct (actual, active; underlying mechanism) would need research and specification. For example, for psychological control, what import does it have in daily life, in certain conditions, e.g., trauma, and what factors buttress it or affect it negatively, e.g., biologically, psychologically (e.g., personality), and socially (e.g., relations, past abuse, if any)? How does the construct work (e.g., through schema, neuronal networks, specifically), and how are these different levels related? Answers to these types of questions add to the validity of the construct, its internal structure and architecture, and the manner in which it mediates and modulates behavior.

To establish whether psychological control is a trauma-specific proximal mechanism, a transdiagnostic construct, or a general adaptation variable, in terms of the paragraph above, psychological control can be considered an actual, active psychological entity that facilitates adaptation to stressors, or deficiencies in this regard, e.g., from either experiencing a perceived overwhelming stressor in the environment and/or a lack in the facility to deal with it. Its efficacy in this regard depends on multiple factors, including whether resilience or psychological reserve is sufficient or optimal, or is insufficient and depleted for the task at hand. It also depends on extant coping skills, problem-solving skills, personality variables, motivation, familial education, family dynamics, partner dynamics, the sociopolitical environment, etc. However, that said, what is the standing of the psychological control in adapting to a perceived environmental stressor? To what degree is it a proximal entity, or a last psychological entity in the serial events physiologically, neuronally, and behaviorally that lead to being overwhelmed by the stressor at hand or not? The articles posit that psychological control is a proximal mechanism in this regard; other mechanisms feed into it, and the collective effect will determine the degree of psychological control experienced in relation to the stressor at hand. Of note, the external stressor has its effects through the perception or appraisal of the stressor by the individual. When the stressor is perceived as overwhelming and taxes the capacity to deal with it, problem solve, maintain physiological equilibrium, etc., the psychological control has a different input into it compared to the opposite case.

Moreover, the stressor at issue does not have to be trauma-specific to activate the adaptive (or maladaptive) mechanism of psychological control. Any perceived stressor that appears overwhelming and taxing the system beyond the person’s limits will activate the psychological control proximal mechanism. In this sense, the mechanism is not transdiagnostic. It is important for any event that is perceived as overwhelming and taxing the system, such as an exceptional loss, a physical injury, a brain affected by a traumatic brain injury, cannabis use, alcohol, etc. It is situationally activated to a dominant protocol in cases of general stress or in cases of specific conditions or diagnoses, such as PTSD, chronic pain, and depression, as well as psychotherapy broadly. It is especially pronounced in cases of overwhelming perceived stressors, for example, and control is mentioned as a factor in the extensive literature review on trauma and PTSD in the article. As well, it is prominent in obsessive–compulsive disorder, given that the condition appears related to a lack of control over the environment.

Indeed, it is posited that psychological control is an actual, active psychological mechanism that is at work constantly and is involved in daily functional adaptation beyond any psychiatric or psychological challenge, condition, or diagnosis. It is the ultimate proximal mechanistic filter to which all input, in one way or another, directly activates or influences it. In this sense, the proximate mechanism of psychological control is a general proximate variable. As well, the associated construct of psychological reserve is a generalized construct that influences directly or indirectly psychological control. Many factors can do the same, either long term ones, such as having experienced child abuse, living in a negative sociopolitical context, etc., or immediate ones, such as fatigue, hunger, pain, poor sleep, feeling isolated, not engaging in self care, not exercising or having hobbies, not socializing, being ill, not being able to find a job, not being able to follow a desired path, such as studying, not being balanced physiologically or psychologically, and so on. Another generalized variable in this regard is internal psychological control, or the ability to modulate internal physiological mechanisms associated with stress response. There are also individual differences, which are external to the context, such as a congenital inability to contain anxiety and panic, leading to elevated adrenaline and cortisol responsivity.

Overall, psychological control can be specified as different from related constructs in that it (a) refers to proximal perception of being overwhelmed by actual contextual stressors; (b) is state-dependent and dynamically modulated by reserve (as well as factors that influence both); and (c) is conceptualized as interacting multiplicatively with reserve (and other factors, but for present mathematical representation and consequent potential empirical testing, the reserve is primary).

### Appendix B.3. The Term

Next, we further provide differential definitions for theory, model, explanation, and mechanism, and indicate how the present theorizing relates to each term. These four terms—model, theory, explanation, and mechanism—represent different, though often overlapping, concepts used in psychological reasoning and research to understand behavior. Theory and modeling are more conceptual, and explanation and mechanism are more causal.

Theory. A comprehensive, abstract framework of principles that explains or predicts a broad range of psychological phenomena. A set of systematically interrelated concepts and propositions that explain a general category of psychological phenomena.

Model. A simplified, concrete, or visual representation used to describe, simulate, or apply specific parts of a psychological theory, such as graphs, that bridges theory and data.

Explanation. Answers “why” a psychological phenomenon occurs by connecting causes to effects. The specific procedure that produces a phenomenon is explained in a step-by-step process.

Mechanism. Describes the “how” of a psychological phenomenon by showing that the phenomenon resulted from a particular process. Provides understanding of the particular causal pathway for observed data.

The present article examines psychological control and psychological reserve from these perspectives. For theory and modeling, they attempt both, and the mathematical representation indicates that they can be structured to be testable empirically. But further specifications are needed with new iterations after empirical testing. For explanation and mechanism, both psychological control and psychological reserve are posited as proximal mechanisms in the serial events leading to behavior after exposure to perceived external stressors. Once more, further specification is needed.

Because this is a theory-driven paper, the typical data engine search procedure does not apply. The concepts were differentiated, and then articles sought to support them in the way indicated. There are hundreds of articles with control as a hit in one way or another, and they were narrowed down to those reviewed.

Including every possible related term to the construct under discussion is the only way of defining boundary conditions for the concept. Also, doing so indicates that all the articles chosen to review are necessary for that purpose. Indeed, we found other articles on psychological control that are reviewed here. Also, we catalog the different concepts related to psychological control to advance the description and differentiation of all these related terms.

Perceived Personal Control. Greenaway (2023) used three items from Greenaway et al. (2015) to measure perceived personal control (“I feel in control of my life”; “I am free to live my life how I wish”; “My experiences in life are due to my own actions”). These items reflect a concept of person control that is more about one’s attitude about oneself in relation to life, rather than a sense of psychological control over the external environment.

Spheres of Control. Nicol (2007) used the Paulhus and van Seltz Spheres of Control Scale (Paulhus & Van Selst, 1990) in their study. It has three separate subscales: personal control, interpersonal control, and sociopolitical control. The latter two scales ostensibly are about the external environment, but inspection of the items reveals this is not the case. The former is about having control in a relationship, and the latter is about people generally having control in the sociopolitical context.

As for grouping into different categories all terms related to psychological control, having the term “control” within them, the terms are psychological control and 15 others: (a) several have components related to psychological control—uncontrollability; sense of control; anxiety control; (b) several relate to whether events are deemed to stem from the self or external to the self—locus of control; internal control; (c) several are about internal factors—fear of losing control; life control; personal control; perceived personal control; overcontrol; (d) several are about external factors—interpersonal control; sociopolitical control; (e) several are about inhibitory control—trait self control; control processes; and (f) compensatory control is a group-based control. Overall, the different categories related to control differentiate from psychological control in the sense of having control of the external environment. Internal psychological control appears related to some of the internal factors involving control, but is more specific as presently defined, and stands in opposition to external psychological control. Overall, the comparative analysis of critical control-related terms supports the uniqueness of the present control concepts and provides clear boundaries differentiating them from other terms in the field of control.

### Appendix B.4. Development

Psychological control is considered a generalized proximal filter in the pathway to stress response to perceived stress. Inevitably, this means that the ability to exert psychological control in order to adapt functionally to external perceived stressors will develop, and the teenager will have less ability to use psychological control effectively, everything else being equal. For example, given that multiple factors can influence it, the psychological reserve that accompanies it, as well as the internal psychological control that leads to physiological expression of the effects of the perceived stressor involved, the adolescent might be lacking in the confluence of factors that lead to its mature and optimal development. The frontal lobes are not fully developed at this age, and the area controls inhibitory skills and their deployment, for example, which could lead to effects on psychological control. Perceiving an external stressor as overwhelming and taxing invokes the question of the cognitive sophistication of the teen’s cognitive architecture and its deployment in the case at hand. Relative to the adult, the teenager might be more vulnerable to appraising a serious external stressor as overwhelming, taxing, traumatic, etc. Beyond that, the neuronal network and linkages with particular brain areas and over them will not be as efficiently differentiated in the adolescent, leading to a less developed neuronal network and architecture that is associated with psychological control. Given the early stage of development of the construct, there has not been research to substantiate these claims, but the lines of future inquiry are at least mapped out in some regards.

Development reflects multiple factors in both the child and the adult. However, the specifics in the two cases can differ, with different emphasis. The younger the child, the more dangerous are biological insults and psychological abuse, for example. At the biological level, the newborn can be quite affected by biological variables, for example. The child’s neurobiological development might suffer long-term impacts from abuse, for example. The teenager might be affected hormonally, for example. As for social factors, the teenager is more susceptible to peer influence, which could impact psychological control. The teenager, psychologically, might have fewer coping skills, generally. As well, at the level of psychiatric medications, particular psychotropics and anti-depressants might be less effective for the adolescent than the adult, leading to disparities in psychological control for the two age groups. Additionally, different versions of psychotherapeutic approaches might have different outcomes for the two age groups. The psychological therapist dealing with adolescents should be cognizant of all these factors that might influence psychological control in adolescents, as well as psychological reserve and internal psychological control. Overall, it is recommended for patients of any age that the therapist consider how the techniques used can help regain psychological control when it is diminished or disturbed. The factors that can do so are biological, psychological, and social, in a combined developmental biopsychosocial model. Moreover, every patient is different in this regard, and the therapist should be attuned to using precision, individualized psychological intervention rather than one approach or more without selecting what might work for the individual patient. Cookie-cutter approaches, no matter how empirically supported, should not be used without adjustment to the person’s uniqueness. That said, only empirically supported treatments should be used for any patient and any condition/diagnosis. The companion article speaks more about this issue. Using a wide-ranging map of possible interventions might appear unwieldy, but using a one-size-fits-all approach is even more potentially damaging to the patient. Finally, because the concepts described herein on psychological control and the depletion of factors that can influence it are a novel approach, the therapist will be using techniques that are empirically supported in psychotherapeutic change mechanism research, but none will have addressed them as involving or related to psychological control/psychological reserve. Until programmatic research is conducted in this vein, the therapist will be left with interpreting whether effective psychotherapeutic techniques for a patient are addressing an underlying psychological control/psychological reserve factor. In the future, psychological control and psychological reserve might be studied psychotherapeutically for their effectiveness in diminishing, removing, preventing relapse, and maintaining gains related to psychological symptoms, diagnoses, conditions, and impairments, and to what degree in the short term and the long term.

#### Theoretical Critique

van Dongen et al. (2026) have described 20 issues related to theory in psychology. We have grouped them under four categories (theory, synthesis, modeling, and empirical work), and respond according to how the present articles have dealt with these issues.

A. On Theory
  1. Most theories are verbal, underspecified, and weakly predictive.
  2. Research data seldom falsify or refine theory.
  3. Constructs are poorly defined, and boundary conditions are not clear.
  4. Constructs are ambiguous, as are auxiliaries, which undermine inference.
  5. Theories are vague and can fit any data, including false positives.
  6. It is difficult to derive hypotheses from theories that are not good.
  7. Imprecise theories make it difficult to make predictions.

The present description of the concepts of psychological control and psychological reserve is very specific with boundary descriptions excluding conceptual ambiguity, overlap, confusion, and inexact understanding and application. The manner in which they might influence behavior is specified. The factors that can influence them are specified. They are represented formally, and simulation studies are run, with each component specified for high vs. low parameterization. The mathematical representation is not vague, but precise and testable (and falsifiable), allowing for specific downstream theory-related hypotheses/predictions, operationalization, research design, and empirical testing. Once the data are collected, the results will permit follow-through with empirical testing. Ensuing research can refine the model and, if disconfirming evidence is obtained, lead to modification of the mathematical representation and further testing. This conceptual and testing interaction will further restrict errors in theory building and promote proper inference, e.g., in what psychological control is and how it can go awry. The validity of the theory will be increasingly supported by ongoing specification of the mathematical representation and subsequent simulation research that functions as a proof of concept, leading to a cycle of empirical testing and model revision.

B. On Synthesis
  8. Theories have little comparison, revision, and updating.
  9. Theories overlap.
  10. Theories survive or “fade away” too slowly.
  11. Incompatible constructs and terms do not foster integration.
  12. Findings are disconnected and do not foster synthesis.
  13. A lack of integration and explanatory connections inhibits unifying theories.
  14. Methodological sophistication is preferred relative to conceptual integration.

The process of concept, model, and theory construction just described above includes a strict mathematical formulation that avoids major issues in specification and allows for empirical investigation of validity. By being so specific and testing the construct, leading to revising and updating, the construct is kept up to date, overlap is avoided, and comparison is facilitated. Indeed, comparison was important in its initial formulation. The theory will avoid hanging around beyond its period of utility by this process, and the original mathematical representation on which it was based. The specificity of the construct helps avoid poor borders with others, incompatibility with others, and removing barriers to integration. For example, the articles include glossaries that clearly indicate the differences with other control concepts, and they could be studied together to determine relative importance in the collection of control concepts and their status in the psychology of the person. Connecting and empirically investigating concepts this way further fosters theoretical integration. With more integrated models, the network profile of the control components in the model can be specified for their linkages, and individual differences can be determined, along with the reasons thereof. The connections might be demonstrated empirically, but determining the underlying mechanisms for them, and the individual differences therein, will require care. Interpretation needs to go beyond empirical results. And, as in any good science, knowledge of biological, psychological, and social factors at play will help. In this way, neither method and data nor concepts and theory are privileged, but they work together in specifying valid and usable models and testable predictions.

C. On Modeling
  15. Formal modeling is underused; it is needed for explicating mechanisms.
  16. Formal models are needed for deriving testable predictions.

As indicated in the two long responses above, the articles under discussion include mathematical representations that have led to proof-of-concept simulation research, preparing for further testing and model revisioning.

D. On Empirical Work
  17. There is a lack of specification for theoretical work vs. empirical research.
  18. Flexible analyses are rewarded.
  19. Measurement and statistics (factor, network) are preferred compared to establishing mechanisms.
  20. Journals and funders seek data-driven articles over theory development and conceptual integration.

The construct is well specified, testable, limits data fishing and fudging to fit the concept, facilitates search for underlying mechanisms, and is open to further theory development and conceptual integration, already having started on the right foot in this regard.
